# Capg enhances proliferation, adipogenesis, and inflammatory response in preadipocytes: insights from bioinformatics analysis and functional validation

**DOI:** 10.7717/peerj.20730

**Published:** 2026-02-10

**Authors:** Luyao Zhang, Botao Sang, Sainan Li, Ying Li, Dachuan Guo, Qinan Ma, Xiangfei Liu, Xiaoshuo Li, Beidong Chen, Deping Liu

**Affiliations:** 1 National Center of Gerontology, Institute of Geriatric Medicine, Chinese Academy of Medical Sciences & Peking Union Medical College, Beijing, China; 2 Beijing Hospital, National Center of Gerontology, Institute of Geriatric Medicine, Chinese Academy of Medical Sciences, Beijing, China; 3 Beijing Hospital, National Center of Gerontology, Institute of Geriatric Medicine, Medical School, University of Chinese Academy of Sciences, Beijing, China; 4 Beijing Hospital, National Center of Gerontology, Chinese Academy of Medical Sciences, Institute of Geriatric Medicine, Beijing University, Beijing, China; 5 The Key Laboratory of Geriatrics, Beijing Institute of Geriatrics, Institute of Geriatric Medicine, Chinese Academy of Medical Sciences, Beijing Hospital/National Center of Gerontology of National Health Commission, Beijing, China

**Keywords:** Adipose tissue, Atherosclerosis, CAPG, Adipogenesis, Inflammation

## Abstract

**Background:**

Various associations between adipose tissue and atherosclerosis (AS) have been revealed. This study aims to identify biomarkers in the epididymal adipose tissue of AS mice and to explore their effects on adipose tissue inflammation and adipogenesis.

**Methods:**

The gene expression profiles of epididymal adipose tissue (GSE57659 and GSE76812) were downloaded from the Gene Expression Omnibus database. Differentially expressed genes (DEG) screened by Limma R package and genes searched by weighted gene correlation network analysis (WGCNA) were performed to classify common genes associated with AS. The Protein-Protein interaction (PPI) network was constructed by Cytoscape software, and hub genes were eventually determined by the Cytohubba plugin. Finally, one of these hub genes was selected. The cell proliferation ability was assessed using the CCK8 assay. Oil Red O staining and Western blot were employed to evaluate the lipid content in adipocytes. The extent of the inflammatory response in adipocytes was determined by Enzyme-Linked Immunosorbent Assay (ELISA).

**Results:**

A total of 125 DEGs were identified between the control group and the atherosclerosis group. Among these, 34 genes were selected based on two key modules identified through WGCNA. Subsequently, five key nodes were identified, namely *Capg*, *Timp1*, *Lgals3*, *Agt*, and *Mmp9*. *Capg* was selected as the primary gene of interest for further investigation. Following the transfection of 3T3-L1 cells with lentivirus, *Capg* was overexpressed. Capping actin protein, gelsolin like (CAPG) significantly enhanced preadipocyte proliferation, as demonstrated by CCK-8 and upregulated expression of the Cyclin D1. Furthermore, Oil Red O staining revealed a marked elevation in intracellular lipid accumulation upon CAPG overexpression. Western blot analysis showed increased protein levels of PPAR γ and adiponectin. Furthermore, CAPG in 3T3-L1 cells resulted in a marked upregulation of IL-6 and MCP-1.

**Conclusion:**

CAPG promotes the proliferation and differentiation of adipocyte precursor cells. Additionally, CAPG enhances the inflammatory response in adipocytes, potentially serving as a key molecule mediating obesity-related atherosclerosis.

## Introduction

Over the past three decades, there has been a significant reduction in cardiovascular mortality.  However, ischemic cardiovascular disease continues to be the leading cause of mortality and morbidity worldwide (Herrington et al., 2016). Atherosclerosis (AS), characterized by the formation of atherosclerotic plaques and infiltration of immune cells, is the primary contributor to cardiovascular diseases (Tsao et al., 2022). Although the etiology and mechanisms underlying AS are complex and not fully understood, chronic low-grade inflammation is observed throughout its progression (Katta et al., 2021). Notably, certain anti-inflammatory therapeutic agents, including colchicine and interleukin-6 (IL-6) inhibitors, have demonstrated significant efficacy in attenuating the progression of atherosclerosis (Nelson, Fuster & Ridker, 2023; Ridker et al., 2021).

Obesity has emerged as a significant risk factor for AS, characterized by the abnormal expansion of adipose tissue (Koskinas et al., 2024). As the largest energy storage organ in the body, adipose tissue plays a central role in regulating glucose and lipid metabolism. Obesity-induced insulin resistance often leads to adipose tissue dysfunction, which exacerbates the progression of atherosclerosis (Liu et al., 2022). Moreover, as an active endocrine organ, hypertrophied adipose tissue communicates with distant tissues *via* paracrine and endocrine, contributing to systemic inflammation and dysregulation of the coagulation and fibrinolytic systems (Sun, Kusminski & Scherer, 2011). Additionally, the secretion of pro-inflammatory adipokines from dysfunctional adipose tissue can induce endothelial dysfunction and accelerate plaque formation, further increasing the risk of AS (Antoniades et al., 2023; Parikh et al., 2009).

Epididymal white adipose tissue (eWAT), a major component of visceral adipose tissue, is critically implicated in the regulation of systemic inflammation (Ohman et al., 2008). To investigate its role in AS, we retrieved two gene expression array datasets of eWAT from AS mice and control mice from the Gene Expression Omnibus (GEO) database. The AS mouse model was established by feeding a high-fat diet to low-density lipoprotein receptor knockout (*Ldlr*^−/−^) mice, a well-established approach to induce atherosclerosis. Through the analysis of differentially expressed genes, we identified the Capping actin protein, gelsolin like (CAPG) as a potential molecular target involved in the progression of AS within eWAT.

## Materials & Methods

### Data collection and preprocessing

Gene expression files were downloaded from the GEO database (http://www.ncbi.nlm.nih.gov/geo/) by using the “GEOquery” package. The datasets GSE57659 and GSE76812 were obtained from eWAT of C57BL/6J male mice with *Ldlr*^−/−^. The GSE57659 expression profile data included 15 samples treated with high-fat diet (HFD, lard 24% w/w) or normal diet (ND) for 16 weeks, while the GSE76812 dataset included 12 samples fed with HFD (with 17.5 kcal% from the cross; D09071704, Research Diets Inc., New Brunswick, NJ, USA) or ND for 20 weeks. The GSE39549 dataset, as the verification dataset, was a bulk RNA-Seq of eWAT from C57BL/6J mice fed ND or HFD for 2, 4, 8, 20, and 24 weeks. The flow diagram of the study is shown in  Supplementary Material 1.

### Identification of differentially expressed gene

The differentially Expressed Gene (DEGs) were determined by the “Limma” package (Version 3.54.2) of R Bioconductor. The adj. *p*-value in our limma-based differential expression analysis is used to correct for multiple hypothesis testing. We used the Benjamini–Hochberg procedure, which controls the False Discovery Rate. Genes with adj. *p*-value < 0.05 and —Log2FC ((fold change)— > 1 were identified as DEGs. The DEGs between the ND group and HFD group were presented in volcano plots based on the ggplot2 R package. A Venn diagram was applied to identify the common DEGs among the GSE76812 and the GSE57659.

### Weighted gene co-expression network analysis

Weighted gene co-expression network analysis (WGCNA) enables the identification of disease-associated modules, thereby greatly improving the discovery of key genes. A hierarchical clustering dendrogram was constructed to delineate gene network modules, with a minimum threshold of 30 genes per module. Module Membership (MM) was defined based on the correlation between the Module Eigenvalue (ME) and the gene expression profiles for each module. Gene Significance (GS) was employed to assess the relationship between individual genes and AS. Utilizing MM and GS, all genes were categorized into several modules, revealing a strong correlation among the genes within each module.

### PPI network construction and screening of hub genes

Common genes derived from DGEs and WGCNA were submitted to the STRING online platform (https://cn.string-db.org/) to construct a Protein-Protein interaction (PPI) network. The CytoHubba plugin in Cytoscape was utilized to identify signature gene clusters and to determine the top five hub genes.

### Cell culture and treatment

The Mouse fibroblast 3T3-L1 cells line (CL-0006, Procell, Bethel, CT, USA) were cultured with Dulbecco’s Modified Eagle’s Medium- High Glucose (DMEM, C11995500BT, Gibco, Waltham, MA, USA) containing 10% fetal bovine serum (FBS, 10091155, Gibco, Waltham, MA, USA) at 37 °C in an incubator containing 5% CO2. When the cells reached 100% confluence, they were cultured with 10% FBS, 0.5 mM 3-isobutyl-1-methyl-xanthine (I5879, Sigma, St. Louis, MO, USA), one µg/ml insulin (I8040, Solarbio, Beijing, China), one µM dexamethasone (D6950, Solarbio), and two µM rosiglitazone (IR0130, Solarbio, Beijing, China) for 48 h. Then, the medium was replaced with DMEM-High Glucose containing 10% FBS, one µg/ml insulin, and two µM rosiglitazone for another 48 h. Subsequently, DMEM-High Glucose containing 10% FBS was maintained for 48 h. Finally, it was treated with 400 µM palmitic acid (Solarbio, Beijing, China) for 48 h.

### Stable overexpression cell line establishment

*Capg* overexpression lentivirus was purchased from Vigene (Biosciences). Twenty-four hours before transfection, 3T3-L1 cells were inoculated into 6-cm dishes with 1 × 10^5^ cells per dish. Twenty-four hours later, overexpression (OE) and control (Ctrl) lentivirus were added separately according to MOI = 100. After 48 h of incubation in the incubator, four µg/ml of puromycin (ST551, Beyotime, Beijing, China) was added to the medium to screen for stably transient cell lines.

### CCK8

According to the manufacturer’s protocol for the CCK-8 assay (C0038, Beyotime, Beijing, China), 3T3-L1 cells were seeded in 96-well plates at a density of 2,000 cells per well and cultured for 48 h. Subsequently, 10 µL of CCK-8 working solution was added to each well, followed by 4 h of incubation in a cell culture incubator. The optical density (OD) at 450 nm was measured using a microplate reader to quantitatively assess cellular viability.

### Lipid quantification

Oil Red O staining was performed to evaluate the degree of lipid accumulation according to the manufacturer’s instruction (G1262, Solarbio, Beijing, China). In summary, cells were fixed with 4% formaldehyde, subsequently stained with Oil Red O solution for 30 min, and then washed before being imaged under a microscope (BZ-X, Keyence). Subsequently, the cells were dissolved in isopropanol, and the absorption peak at a wavelength of 520 nm was measured using a spectrophotometer.

### Western blot

Adipocytes were collected and disrupted in the protein lysate, including RIPA buffer (R0010, Solarbio, Beijing, China) with 1% phosphatase inhibitor (P1260, Solarbio, Beijing, China) and 1% PMSF (P0100, Solarbio). The protein concentration of the samples was determined using a BCA protein assay kit (23225, Pierce™ BCA Protein Assay Kits). Electrophoresis was performed on a 10% polyacrylamide gel to separate the proteins. The proteins on the gel were subsequently transferred onto a PVDF membrane (ISEQ00010, Millipore, Burlington, MA, USA). The membrane was closed with 5% skimmed milk for 120 min at room temperature. The membrane was cut to the appropriate size according to the molecular weight of the target protein and incubated overnight at 4 °C with the corresponding primary antibody. Primary antibodies used in this study included CAPG (1:2000, 10194-1-AP, Proteintech, Rosemont, IL, USA), β-tubulin (1:6000, 10094-1-AP, Proteintech, Rosemont, IL, USA), DDDDK-Tag (ABclonal, AE169PM, 47kD), PPARγ (1:1000, #2435T, CST), Adiponectin (1:1000, #2789, CST), CylinD1(1:2000, HY-P80098, MCE). Subsequently, HRP-conjugated goat anti-rabbit IgG antibody (1:5000, #7074, CST) was diluted with 5% skimmed milk powder (P0216, Beyotime, Shanghai, China) and incubated at room temperature for 1 h, followed by followed by densitometric analysis using the Image J software.

### ELISA

In accordance with the manufacturer’s protocol, the cell supernatant was collected, and the levels of IL-6(ABclonal, RK00008, China), TNF-α (ABclonal, RK04875, China), and MCP-1(ABclonal, RK00381, China) were quantified in both groups of cell samples.

### Statistical methods

Statistical analyses were performed using GraphPad Prism 8.0 software (GraphPad Software). Intergroup comparisons of normally distributed data were conducted using a two-tailed unpaired Student’s *t*-test. A *p* value of less than 0.05 was considered statistically significant.

## Results

### Common differentially expressed genes

**Figure 1:**
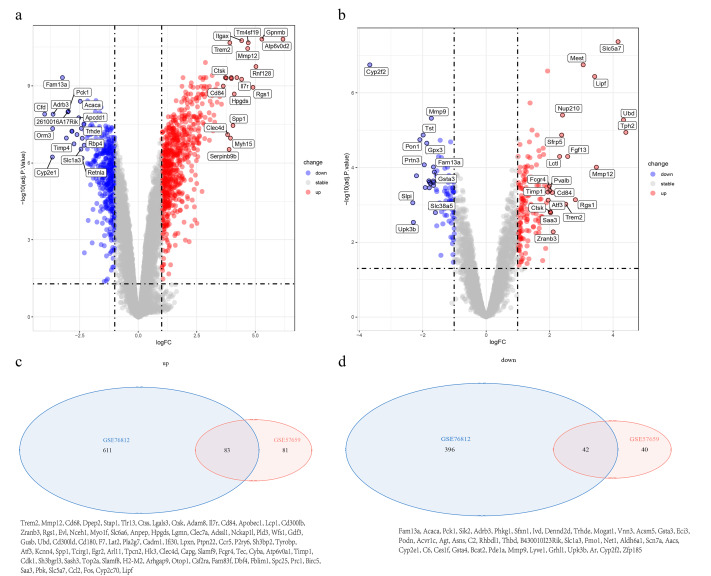
Identification and overlap of differentially expressed genes (DEGs) in atherosclerotic datasets. (A, B) Volcano plots visualizing DEGs in the (A) GSE76812 and (B) GSE57659 datasets. Genes with statistically significant up-regulation (red) and down-regulation (blue) were identified using thresholds of |log2 fold change| > 1 and adjusted *p*-value (FDR) < 0.05. Grey dots represent non-significant genes. The top 20 significantly dysregulated genes are labeled. (C, D) Venn diagrams illustrating the overlap of (C) up-regulated and (D) down-regulated DEGs between the two datasets. The intersection reveals a core set of 83 consistently up-regulated and 42 consistently down-regulated genes across independent studies, highlighting robust transcriptional alterations in atherosclerosis.

We analyzed the two datasets separately, filtered the corresponding DEGs, and presented them in the form of volcano plots. In the GSE76812 dataset, there were 1,132 DEGs, including 694 up-regulated genes and 438 down-regulated genes (Fig. 1A). In the GSE57659 dataset, 246 genes were clarified as DEGs, of which 164 genes were up-regulated and 82 genes were down-regulated (Fig. 1B). We then took the intersection of the DEGs of the two datasets and used Veen diagrams to show the common 83 up-regulated and 42 down-regulated DEGs (Figs. 1C–1D).

### Key genes from WGCNA

**Figure 2:**
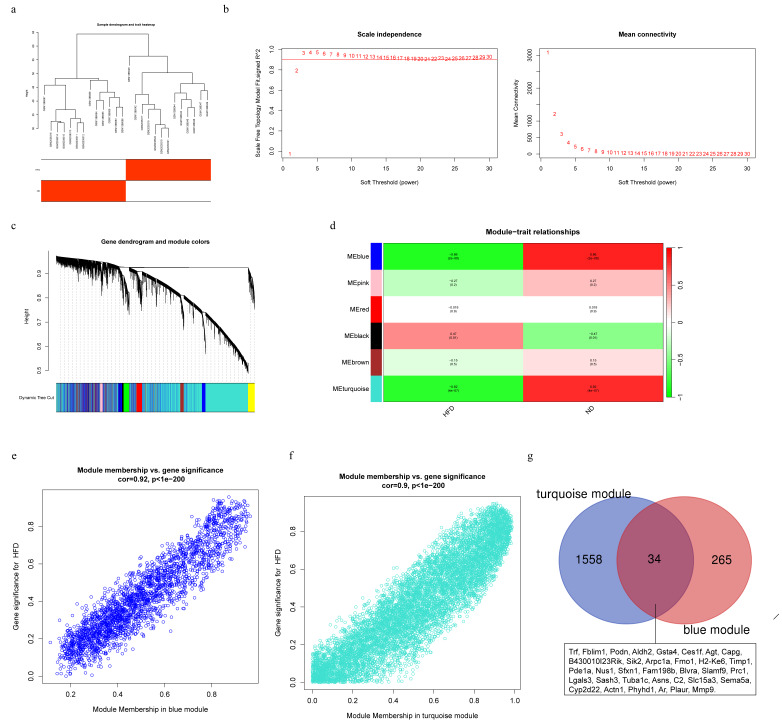
Weighted gene co-expression network analysis (WGCNA) identifies key modules associated with high-fat diet. (A) Sample clustering dendrogram. One outlier sample was identified and excluded from subsequent network construction to ensure robustness. (B) Analysis of network topology for various soft-thresholding powers. The left panel shows the scale-free topology fit index (*R*^2^), and the right panel shows the mean connectivity. The selected power of β = 3 was the lowest power at which the network approximated a scale-free topology (*R*^2^ > 0.9). (C) Hierarchical clustering dendrogram of genes. Genes are grouped into modules of highly co-expressed genes, with each branch and color representing one module. (D) Module-trait associations heatmap. Each row corresponds to a gene module, and each column corresponds to a clinical trait. The color intensity represents the correlation coefficient (from −1 to 1), with associated *p*-values in parentheses. The blue and turquoise modules showed the strongest significant positive correlation with the HFD trait. (E, F) Scatterplots of Gene Significance (GS) for HFD *versus* Module Membership (MM) in the (E) blue (correlation = 0.92, *p* < 1*e* − 200) and (F) turquoise (correlation = 0.9, *p* < 1*e* − 200) modules. The strong positive correlations indicate that genes highly significant for HFD are also central elements within their respective modules. (G) Venn diagram showing the overlap of genes between the blue and turquoise modules, revealing 34 common genes that may represent a core transcriptional response to HFD.

Following the identification of an outlier sample by sample cluster analysis, a total of 26 samples were clustered (Fig. 2A). In this study, a soft-threshold power of 3 was selected, achieving a scale-free R^2^ of 0.9 (Fig. 2B). Ultimately, six distinct modules were identified, each represented by different colors (Figs. 2C, 2D). Correlation analysis between these modules and clinical traits revealed that the blue module (Cor = −0.86, *P* = 2*e* − 8), turquoise module (Cor = −0.82, *P* = 4*e* − 7), and black module (Cor = 0.47, *P* = 0.01) exhibited significant associations with HFD. Notably, the black module was positively correlated with HFD, while the blue and turquoise modules were negatively correlated with HFD. Given the strong correlation between the blue and turquoise modules, we applied the filtering criteria of |MM| > 0.8 and |GS| > 0.20, resulting in the identification of 299 genes in the blue module and 1592 genes in the turquoise module (Figs. 2E, 2F). Figure 2G highlights the 34 common genes shared between these two key modules.

### Identification of hub gene

**Figure 3:**
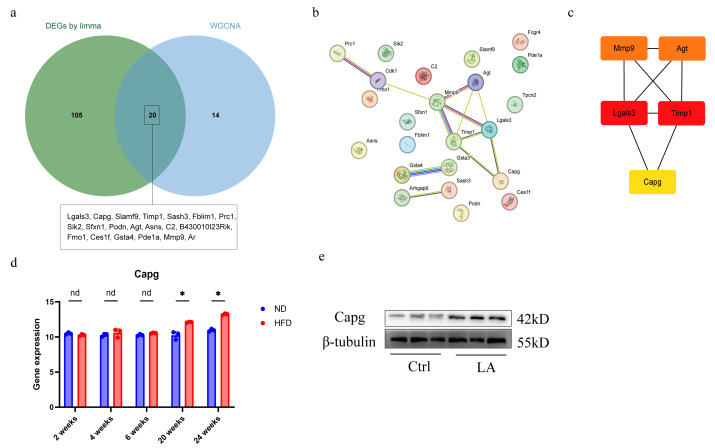
Identification and validation of *Capg* as a key hub gene in adipose dysfunction. (A) Venn diagram illustrating the overlap between differentially expressed genes (DEGs) and genes from the key WGCNA modules, identifying a core set of candidate hub genes for further investigation. (B) Protein-protein interaction (PPI) network of the common hub genes identified in (A). Nodes represent proteins, and edges represent predicted functional associations. (C) The top five hub genes ranked by connectivity within the PPI network, as identified by the CytoHubba plugin. (D) Independent validation of CAPG mRNA expression in the GSE39549 dataset. CAPG levels were significantly upregulated in the HFD group compared to the ND group (*p < 0.05). The label “nd” (not determined) refers to samples where data was unavailable. (E) Western blot analysis confirming the upregulation of CAPG protein expression in differentiated mature adipocytes treated with 400 µM linoleic acid (LA) for 48 h, compared to the vehicle control.

To identify hub genes, we performed an intersection of the common DEGs and the key genes identified through WGCNA (Fig. 3A). The PPI network illustrates the key nodes and their interactions among the DEGs, while the cytoHubba plugin identifies the top five hub genes based on the maximum connectivity criterion (MCC) algorithm score (Figs. 3B–3C). We selected CAPG for further study as it was previously found upregulated in adipose tissue of HFD-fed mice, while its specific function in adipose tissue during atherosclerosis remains largely unknown, presenting a significant knowledge gap to address.

To eliminate the potential confounding effects of *Ldlr*^−/−^ on the expression of Capg, we utilized RNA-Seq data from wild-type C57BL/6J mice subjected to ND and HFD treatments, sourced from the GEO. As illustrated in Fig. 3D, there were no significant differences in the expression levels of *Capg* in the eWAT between the two groups at 2, 4, and 8 weeks of treatment. However, after 20 and 24 weeks of HFD and ND treatments, the mRNA level of *Capg* in the HFD group was significantly elevated compared to that in the ND group. After treating differentiated mature adipocytes with the vehicle and linoleic acid (LA), Western blot results indicated a significant increase in CAPG expression levels (Fig. 3E).

### CAPG promoted proliferation of 3T3-L1 cells

**Figure 4:**
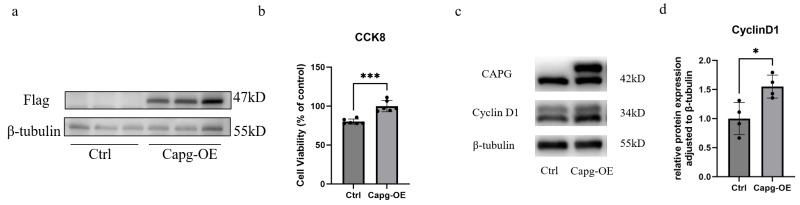
Overexpression of CAPG enhances the proliferation of 3T3-L1 preadipocytes. (A) Western Blot analysis confirming the successful overexpression of CAPG in 3T3-L1 cells transduced with CAPG-encoding lentivirus (*Capg*-OE) compared to control lentivirus (Ctrl). The membrane was probed with an anti-Flag antibody. (B) Cell viability measured by CCK-8 assay over 2 *days*. CAPG overexpression significantly promoted cell proliferation compared to the control group (*n* = 6 biological replicates per group). Data are presented as mean ± SD. (C) Representative Western Blots showing protein levels of the proliferation marker Cyclin D1 and CAPG in Ctrl and *Capg*-OE groups. (D) Quantitative densitometric analysis of the Western Blots from (C) (*n* = 4 independent experiments). CAPG overexpression led to a significant upregulation of Cyclin D1 protein levels, consistent with the enhanced proliferation phenotype. Data in (B) and (D) were analyzed by Student’s *t*-test. * *p* < 0.05; *** *p* < 0.001.

To investigate the effect of CAPG on the proliferation of 3T3-L1 cells, we conducted CCK-8 assays on both Ctrl and *Capg*-OE cells. The results demonstrated that CAPG significantly enhanced the proliferation of 3T3-L1 cells (Fig. 4B). Subsequently, we assessed the expression of the cell cycle-related protein CyclinD1 and found that CAPG significantly upregulated CyclinD1 levels in 3T3-L1 cells (Figs. 4C–4D).

### CAPG promoted adipogenesis during preadipocyte differentiation

**Figure 5:**
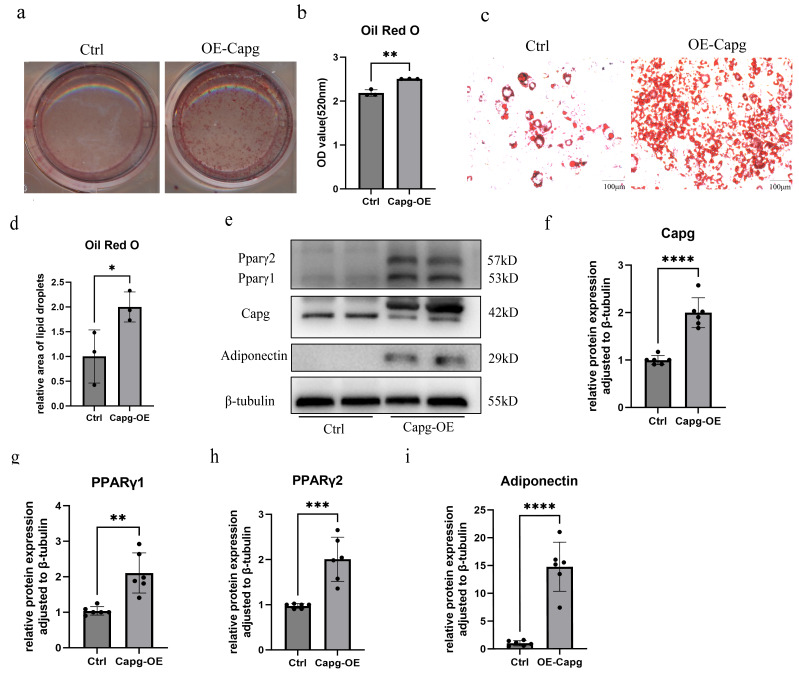
CAPG overexpression promotes adipogenesis in 3T3-L1 cells. (A) Representative images of Oil Red O staining in control (Ctrl) and CAPG-overexpressing (*Capg*-OE) adipocytes on day eight of differentiation, visualizing lipid accumulation. (B) Quantitative analysis of the Oil Red O staining shown in (A) by elution and spectrophotometry (*n* = 3 independent experiments). (C) High-magnification (200×) microscopic fields of the Oil Red O staining from (A); scale bar = 100 µm. (D) Quantitative analysis of the lipid droplet-positive area from the microscopic images in (C) (*n* = 3 independent experiments). (E) Representative Western Blots analyzing the protein levels of CAPG and key adipogenic markers (PPARγ, adiponectin) in Ctrl and Capg-OE cells. (F–I) Densitometric quantification of the protein levels from (E) for (F) CAPG, (G) PPARγ, (H) PPARγ2, and (I) adiponectin (*n* = 6 independent experiments). Data are presented as mean ± SD. Statistical significance was determined by Student’s *t*-test. **p < 0.01; ***p < 0.001; ****p < 0.0001.

To evaluate the role of *Capg* in adipogenesis, we induced differentiation in cells from both the Ctrl group and the *Capg*-OE group. On day 10 of differentiation, we performed Oil Red O staining on the adipocytes from both groups. As illustrated in Fig. 5A, CAPG significantly promotes the differentiation of preadipocytes into mature adipocytes. We extracted lipids from the Oil Red O-stained cells using isopropanol and quantified the resulting extract at a wavelength of 520 nm. The results demonstrated that the absorbance at 520 nm in the *Capg*-OE group was markedly higher than that in the Ctrl group (Fig. 5B). Additionally, microscopic examination of the Oil Red O staining revealed a significantly greater number of positively stained cells in the *Capg*-OE group compared to the control group (Figs. 5C–5D). Western Blot analysis further confirmed that CAPG significantly enhances the expression of PPARγ and adiponectin (Figs. 5E–5I), which aligns with the staining results previously mentioned.

### CAPG exacerbated inflammatory responses in adipocytes

**Figure 6:**
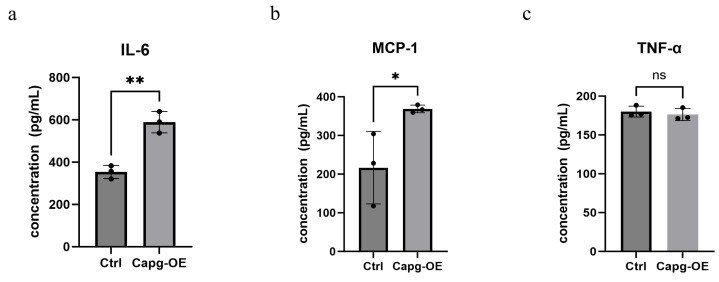
CAPG overexpression selectively promotes the secretion of specific pro-inflammatory cytokines. (A–C) Concentrations of pro-inflammatory cytokines (A) IL-6, (B) MCP-1, and (C) TNF-α in the cell culture supernatant of control (Ctrl) and CAPG-overexpressing (*Capg*-OE) 3T3-L1 adipocytes, as measured by ELISA. Data are presented as mean ± SD from three independent experiments (*n* = 3). Statistical significance was determined by Student’s *t*-test. *p < 0.05; **p < 0.01; ns, not significant.

We evaluated the expression levels of inflammatory factors in both adipocytes and the supernatants from the Ctrl and *Capg*-OE groups. Our results demonstrated that *Capg*-OE significantly increased the concentrations of IL-6 and MCP-1 in the adipocyte supernatants (Figs. 6A–6C).

## Discussion

In this study, we analyzed two microarray datasets from eWAT of *Ldlr*^−/−^ mice subjected to ND and HFD. Through comprehensive bioinformatics analysis, *Capg* was identified as a pivotal hub gene in the eWAT of AS mice. Functional studies demonstrated that CAPG in 3T3-L1 preadipocytes significantly enhanced proliferation and adipogenesis. Further, CAPG upregulated the secretion of pro-inflammatory cytokines, including IL-6 and MCP-1. These findings suggest a critical role of CAPG in regulating adipogenesis and inflammation in the context of diet-induced metabolic dysregulation.

An increasing body of research has identified crosstalk between adipose tissue and blood vessels, suggesting that ectopic adipose deposition may play a critical role in exacerbating AS progression (Subramanian et al., 2008). Specifically, chemokines, adipokines, and inflammatory factors could reach the vascular endothelium, mediating endothelial damage and the infiltration of inflammatory cells (Ahmadieh, Kim & Weintraub, 2020; Farias-Itao et al., 2022). To investigate this further, we conducted a bioinformatics analysis of DEGs in eWAT from mice subjected to HFD compared to those on ND, aiming to identify the hub gene associated with adipose tissue dysfunction. It is important to note that LDLr primarily facilitates the transport of LDL *in vivo*. When LDLr is knocked out, adipocyte differentiation and inflammation may be adversely affected due to diminished LDL uptake (Farias-Itao et al., 2022). To mitigate the potential influence of LDLr knockout on our findings, we assessed *Capg* gene expression in the eWAT of wild-type mice. Our results revealed that the upregulation of *Capg* correlated with the duration of HFD treatment, with significant elevation in *Capg* expression levels only observed after 20 weeks of HFD exposure. This suggests that the upregulation of *Capg* may occur in the later stages of obesity.

CAPG, a member of the gelsolin protein family, was initially identified for its role in macrophages, and subsequent studies have revealed its expression across various tissues and cell types (Dabiri et al., 1992). Similar to other proteins within the gelsolin superfamily, CAPG plays a critical role in the regulation of cell migration, proliferation, and other biological processes through the organization of actin filaments in non-myocytes (Silacci et al., 2004). However, it is distinct in that it solely possesses the ability to bind to actin, lacking shear activity; thus, it is specifically involved in the regulation of fibroblast and endothelial cell motility. Numerous studies have reported elevated CAPG expression levels in atherosclerotic blood vessels (Li et al., 2015; Shen et al., 2021). Notably, the rs6886 polymorphism in the CAPG gene has been significantly associated with carotid intima-media thickness. Hansson et al. (2006) observed increased CAPG expression in white adipose tissue from wild-type mice subjected to an HFD. In our study, we also demonstrated that *Capg* expression was elevated in adipose tissue in the HFD group, which may be attributed to heightened levels of HIF-1α induced by hypoxic conditions. Previous research indicates that CAPG is a target protein of HIF-1α, and its expression can be specifically inhibited by short hairpin RNA targeting HIF-1α (Liao et al., 2009).

Research has shown that the number of preadipocytes increases during the process of obesity (Jeffery et al., 2015), a change that may provide a foundation for the expansion of adipose tissue. In this study, CAPG in 3T3-L1 cells enhanced their proliferative capacity. Previous studies have highlighted the proliferative and migratory capabilities of CAPG, particularly within the context of cancer (Liu et al., 2024; Wang et al., 2022). Notably, the silencing of *Capg via* siRNA has been shown to substantially inhibit the proliferation of DU145 cells (Li et al., 2015). Long et al. (2024) reported that CAPG promotes the proliferation of gastric cancer cells by activating the Wnt/β-catenin signaling pathway. Our findings revealed that CAPG significantly enhanced the expression of CyclinD1, thereby facilitating the proliferation of 3T3-L1 cells.

In addition to increasing the number of preadipocytes, CAPG significantly enhanced adipogenic differentiation capacity. The expression of adipogenesis-related genes, including *Ppar*γ, *Fasn*, *Fabp4*, and *Adipoq*, was markedly diminished in the eWAT of mice subjected to an HFD (Neuhofer et al., 2014). Conversely, our results indicate that CAPG significantly promotes adipogenesis, which is beneficial for alleviating AS. This finding is consistent with previous research by Wang et al. (2013) who demonstrated that an HFD promotes the differentiation of preadipocytes into mature adipocytes, a process referred to as hyperplasia. The underlying cause of this discrepancy remains unclear. On one hand, it may be associated with variations in the duration of high-fat diet treatment. Studies have demonstrated that early-stage obesity primarily involves adipocyte hypertrophy, and once adipocytes reach their maximum size, they may stimulate the generation of new adipocytes (Cleary, Brasel & Greenwood, 1979; Faust et al., 1978). On the other hand, CAPG might enhance differentiation capacity by increasing the population of preadipocytes, representing a potential compensatory mechanism.

Visceral adipose tissue inflammation is closely associated with adipocyte dysfunction and serves as a significant risk factor for systemic metabolic disturbances, including dysregulation of lipid and glucose homeostasis (Le Jemtel et al., 2018). CAPG significantly upregulates the expression levels of inflammatory factors. The NF-κB signaling pathway is a crucial regulator of the inflammatory response in adipose tissue (Xu et al., 2024). Ma et al. (2023) have reported a correlation between CAPG and this pathway. They utilized mass spectrometry-based immunoprecipitation to purify and characterize the CAPG protein complex in the human acute myeloid leukemia cell line THP-1. Through this analysis, they identified interactions between CAPG and NF-κB-related proteins, including RPL4, ZFP91, and CCAR2, all of which are known activators of the NF-κB signaling pathway. Further, as a key regulator of actin cytoskeleton dynamics, it is plausible that the phenotypic consequences of CAPG overexpression are initiated through cytoskeletal remodeling. The actin cytoskeleton is not merely a structural scaffold but also a dynamic signaling platform that influences diverse cellular processes, including mechanotransduction, vesicle trafficking, and the activation of transcription factors such as NF-κB and JNK signaling pathways (Nennig & Schank, 2017; Phusuntornsakul et al., 2020). Therefore, the upregulation of inflammatory cytokines (*e.g.*, IL-6, MCP-1) and adipogenic markers observed in our study could be a downstream consequence of CAPG-driven alterations in cell shape, stiffness, or mechanical signaling.

Interestingly, while CAPG overexpression robustly elevated the levels of IL-6 and MCP-1, it did not significantly alter TNF-α expression. A potential explanation for this differential secretion profile is that CAPG may exert dual functions in adipocytes. On one hand, CAPG overexpression may activate specific pro-inflammatory signaling pathways (*e.g.*, potentially involving NF-κB or other cascades) that preferentially drive the production and release of IL-6 and MCP-1. On the other hand, Ohkawa et al. (2023) demonstrated that CAPG functions as a transcriptional repressor by directly binding to the promoter of TNF-α, thereby inhibiting its expression. It is plausible that in adipocytes, this repressive function concurrently suppresses the expression or secretion of TNF-α. This opposing action could result in the net outcome we observed: a significant increase in IL-6 and MCP-1 alongside a non-significant change in TNF-α. Elucidating the precise molecular mechanism underlying this selective cytokine regulation indeed requires more in-depth experimental investigation in future studies.

Although CAPG promotes the increase in the number of preadipocytes and mature adipocytes, it significantly enhances the expression of inflammatory factors in the adipocyte supernatant, thereby accelerating the progression of AS. Therefore, CAPG may serve as a key therapeutic target for modulating obesity-related adipose tissue dysfunction and AS. However, this study still has several limitations. Firstly, although the hub gene, *Capg*, was identified through comprehensive bioinformatics analysis, its expression was validated only within the dataset and *in vitro* models, lacking corroboration through *in vivo* animal experiments. Secondly, the investigation was confined to the adipogenesis of preadipocytes, leaving the potential effects of *Capg* on mature adipocytes unexplored and necessitating further research. Thirdly, the precise molecular mechanisms underlying *Capg*-mediated regulation of inflammatory responses and adipogenesis remain to be elucidated, representing a critical area for future studies. Lastly, our findings are primarily derived from gain-of-function approaches, we cannot yet conclusively state that CAPG is necessary for these processes under physiological or pathological conditions. Future studies employing loss-of-function strategies, such as RNA interference or CRISPR/Cas9-mediated knockout of CAPG in relevant *in vitro* and *in vivo* models, will be essential to definitively establish its necessity and further elucidate its mechanistic role. Addressing this point will be a critical focus of our ongoing research.

## Conclusions

In conclusion, this study successfully identified *Capg* as a pivotal gene associated with high-fat diets in visceral adipose tissue through bioinformatics analysis and validated its effects on proliferation, adipogenesis and inflammation *in vitro*. Future research should aim further to clarify the role of CAPG in adipocyte biology and explore its potential implications for metabolic homeostasis.

##  Supplemental Information

10.7717/peerj.20730/supp-1 Supplemental Information 1 Bioinformatics Workflow for the Identification of Hub Genes Associated with Atherosclerosis in Mouse Epididymal White Adipose Tissue (eWAT) The flowchart outlines the stepwise strategy for screening hub genes. The analysis commenced with the acquisition of transcriptomic datasets GSE76812 and GSE57659. Key steps included the identification of Differentially Expressed Genes (DEGs), the construction of a co-expression network using WGCNA to identify key modules, and the integration of these results to obtain common genes. These genes were then used to construct a Protein-Protein Interaction (PPI) network, from which hub genes were finally filtered based on connectivity scores using the CytoHubba plugin. Ultimately, the gene of interest was identified and its differential expression was validated in the GSE39549 dataset and through in vitro cell experiments.

10.7717/peerj.20730/supp-2 Supplemental Information 2 Raw Western Blots data All groups and samples are circled with red boxes and labeled with specific information in the figures.

10.7717/peerj.20730/supp-3 Supplemental Information 3 Full set of representative images supporting the quantitative analysis of lipid accumulation The complete visual evidence for the Oil Red O staining analysis presented in the main text. The images were captured following a standardized protocol (100× magnification, five random fields per well) to ensure unbiased assessment. Supplementary Figure S1 provides a macroscopic, plate-level overview, clearly illustrating the enhanced lipid accumulation in the CAPG-overexpressing (*Capg*-OE) groups compared to the controls (Ctrl) across all replicates. Supplementary Figures S2-S4 present the negative control baseline, showing three independent replicate wells from the Ctrl group. These figures demonstrate the consistency and low level of adipogenesis in the absence of CAPG overexpression. Supplementary Figures S5-S7 present the experimental results, showing three independent replicate wells from the *Capg*-OE group. The uniformly intense staining across all *Capg*-OE replicates provides robust and reproducible visual evidence of the pro-adipogenic effect of CAPG.

10.7717/peerj.20730/supp-4 Supplemental Information 4 Complete dataset for functional validation experiments The raw quantitative data and statistical calculations for all key functional assays presented in the manuscript. The data is organized into separate sheets as detailed below. Sheet 1: CCK-8 Assay:Raw absorbance values (450 nm) and calculated cell viability/proliferation data for Control (Ctrl) and CAPG-overexpression (*Capg*-OE) groups over a time course. Sheet 2: Oil Red O Quantification:Raw data from the elution and spectrophotometric measurement of Oil Red O stain, quantifying total lipid content in differentiated adipocytes. Sheet 3: Oil Red O Positive Area Analysis (Microscopy):Quantitative image analysis data measuring the area of lipid droplets (Oil Red O positive regions) from multiple microscopic fields. Sheet 4-6: ELISA for Inflammatory Cytokines:Raw concentration values (pg/mL) for the pro-inflammatory cytokines MCP-1(Sheet 4), IL-6(Sheet 5), and TNF-α (Sheet 6) measured in cell culture supernatants. All statistical comparisons between the Ctrl and *Capg*-OE groups were performed using a two-tailed Student’s t-test. Data are presented as mean ± standard deviation (SD).

10.7717/peerj.20730/supp-5 Supplemental Information 5 Code

10.7717/peerj.20730/supp-6 Supplemental Information 6 Translation codebook

## Additional Information and Declarations

### Data Availability

The following information was supplied regarding data availability: The data is available at NCBI GEO: GSE57659, GSE76812, GSE39549 and raw measurements are available in the Supplemental Files.
