# Supplementary figures and images for: Capg enhances proliferation, adipogenesis, and inflammatory response in preadipocytes: insights from bioinformatics analysis and functional validation
Source: PeerJ. 2026 Feb 10;14:e20730. doi: 10.7717/peerj.20730 (PMC12903893; doi:10.7717/peerj.20730)

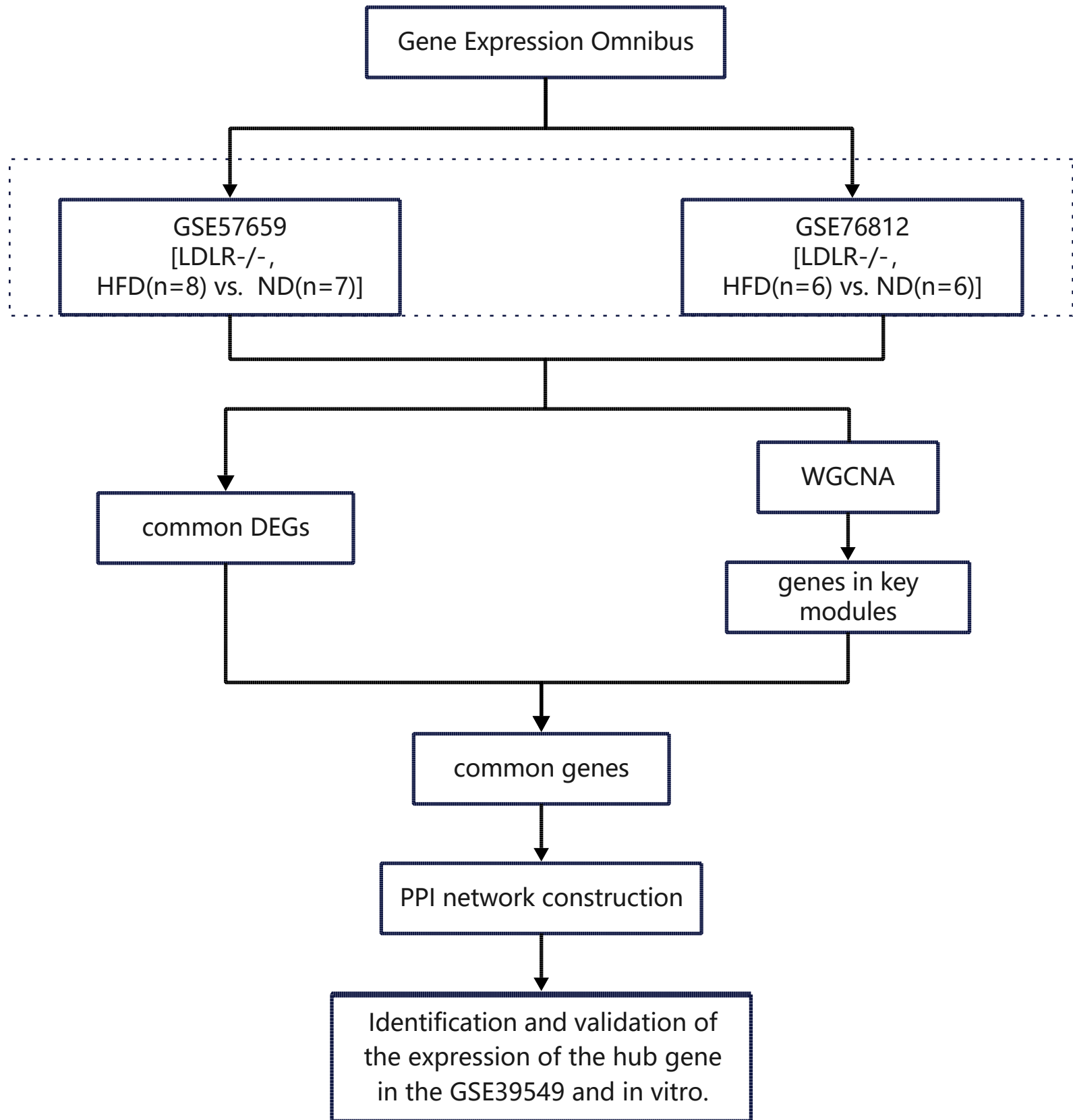

Supplement: Supplemental Information 1 — The flowchart outlines the stepwise strategy for screening hub genes. The analysis commenced with the acquisition of transcriptomic datasets GSE76812 and GSE57659. Key steps included the identification of Differentially Expressed Genes (DEGs), the construction of a co-expression network using WGCNA to identify key modules, and the integration of these results to obtain common genes. These genes were then used to construct a Protein-Protein Interaction (PPI) network, from which hub genes were finally filtered based on connectivity scores using the CytoHubba plugin. Ultimately, the gene of interest was identified and its differential expression was validated in the GSE39549 dataset and through in vitro cell experiments. [file peerj-14-20730-s001.pdf]
