# Supplementary material for: Capg enhances proliferation, adipogenesis, and inflammatory response in preadipocytes: insights from bioinformatics analysis and functional validation
Source: PeerJ. 2026 Feb 10;14:e20730. doi: 10.7717/peerj.20730 (PMC12903893; doi:10.7717/peerj.20730)
Supplement: Supplemental Information 2 — All groups and samples are circled with red boxes and labeled with specific information in the figures. [file peerj-14-20730-s002.docx]

**
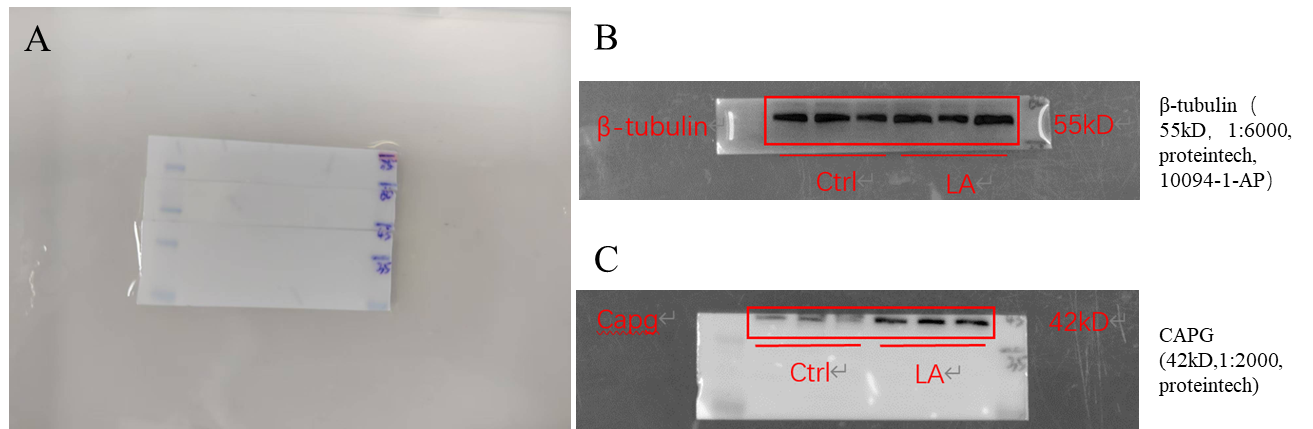
**

**Supplementary Figure S1. Raw Western Blot Images of Western blots shown in Figure 3e**

**(A)** These full, spliced images correspond to the representative blots shown in Figure 3e. **(B)** Membrane probed for β-tubulin (loading control). **Lane 1:** Molecular weight marker. **Lane 2-4:** Control (Vehicle) sample. **Lane 5-7:** 400 μM Linoleic Acid (LA) treated sample. **Lane 8:** Molecular weight marker. **(C)** Membrane probed for CAPG. The red rectangles indicate the regions that were used to construct the final figure. Molecular weights (kDa) are indicated by the marker.

**
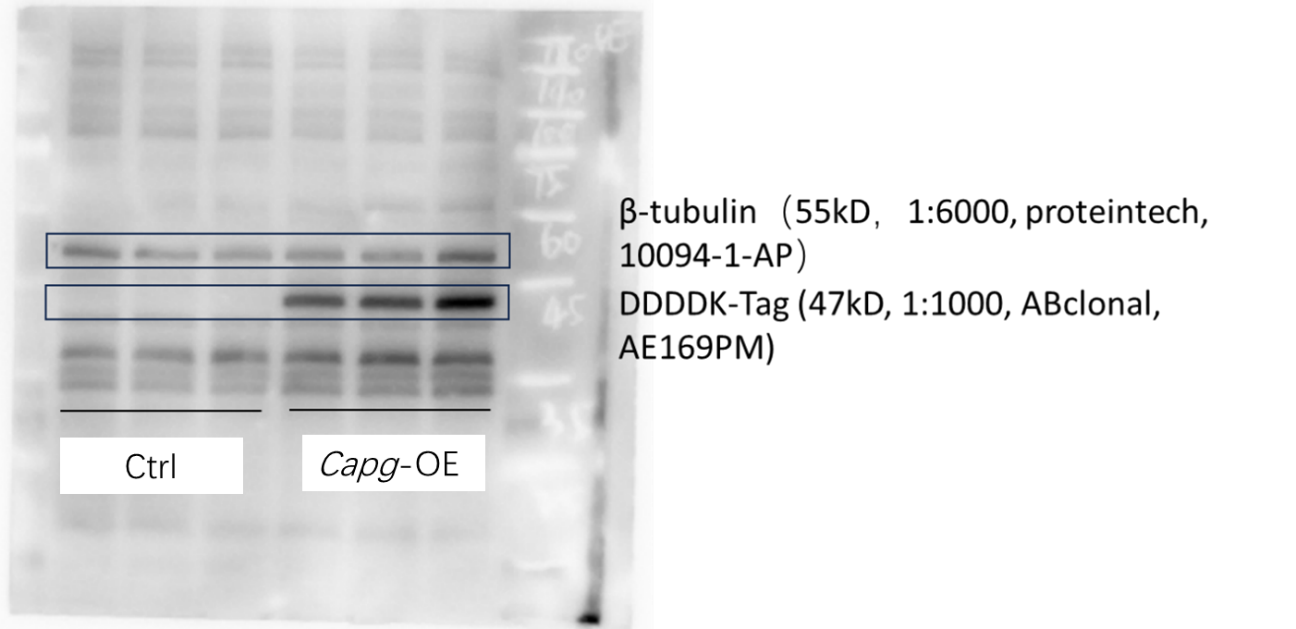
**

**Supplementary Figure S2. Raw Western Blot Images of Western blots shown in Figure 4a**

**(A)**  Uncropped scans of the Western blot membranes presented in Figure 4a. **Lane 1:** Molecular weight marker. **Lane 2-4:** Control (Vehicle) sample. **Lane 5-7:** CAPG-overexpression (*Capg*-OE) sample. **Lane 8:** Molecular weight marker. The blue rectangles indicate the regions that were used to construct the final figure. The upper blue box indicates the area probed with the β-tubulin antibody (loading control), while the lower blue box indicates the area probed with the Flag antibody.

Molecular weights (kDa) are indicated by the marker.


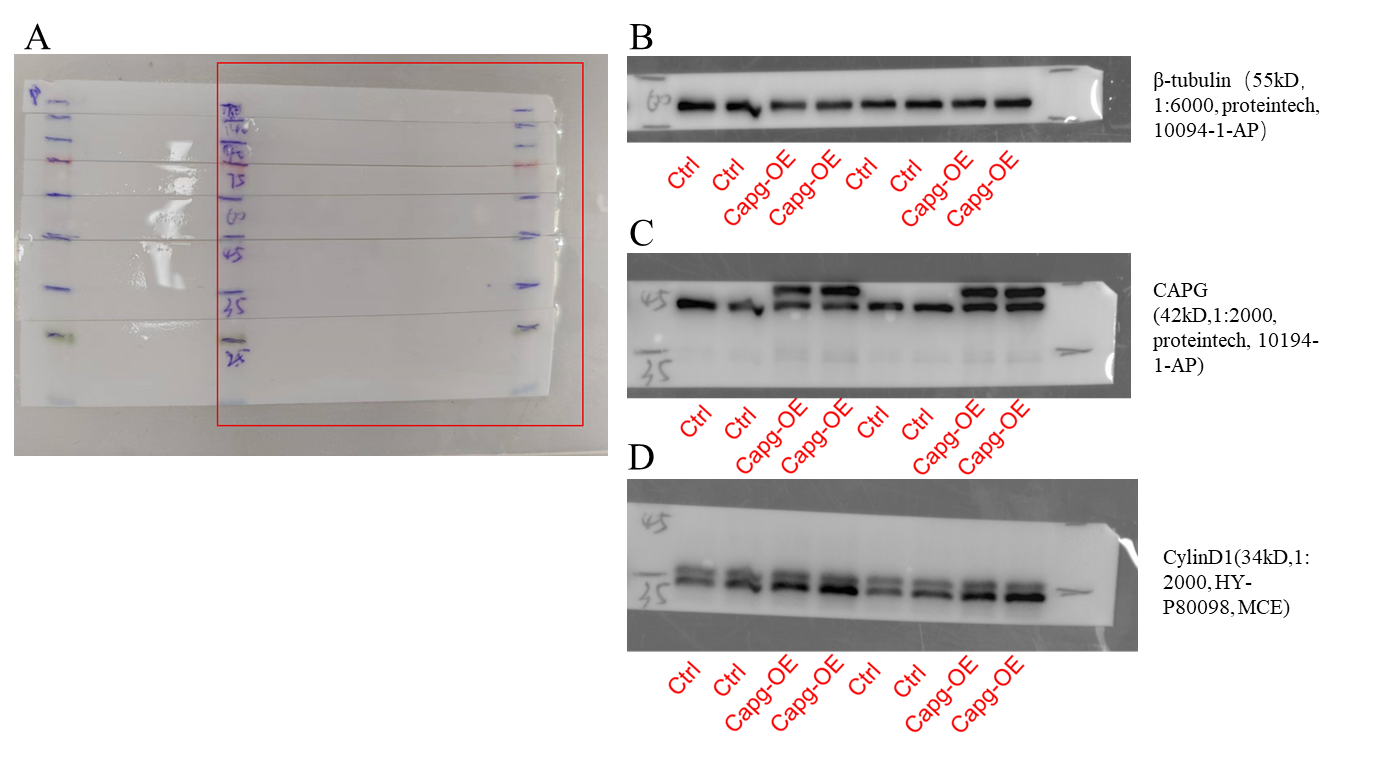


**Supplementary Figure S3. Raw Western Blot Images of Western blots shown in Figure 4c-d**

**(A)** These full, spliced images correspond to the representative blots shown in Figure 4c-d. **(B)** Membrane probed for β-tubulin (loading control). **Lane 1:** Molecular weight marker. **Lane 2-3,6-7:** Control (Vehicle) sample. **Lane 4-5,8-9:** 400 μM Linoleic Acid (LA) treated sample. **Lane 10:** Molecular weight marker. **(C)** Membrane probed for CAPG. **(D)** Membrane probed for Cyclin D1.


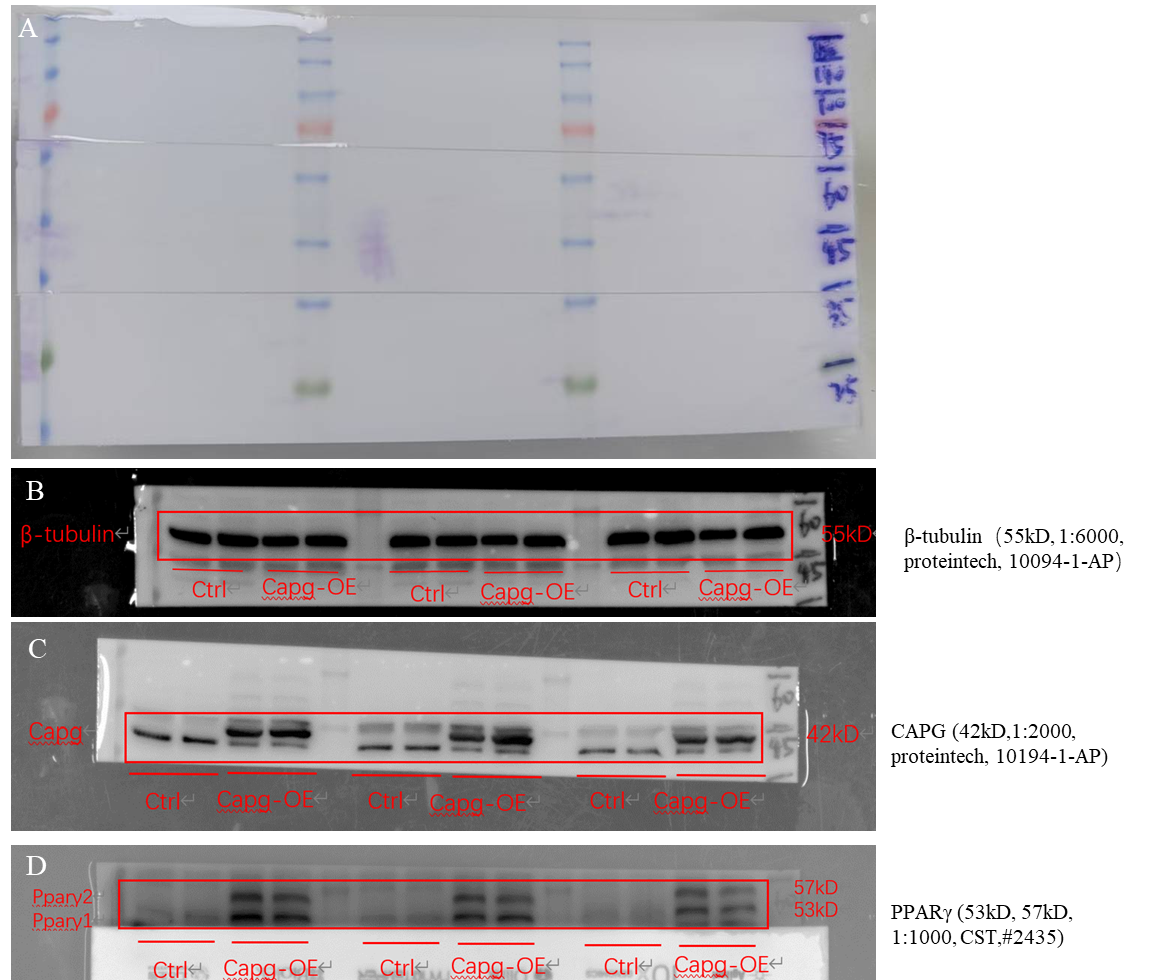


**Supplementary Figure S4. Raw Western Blot Images of Western blots shown in Figure 5e-h**

**(A)** These full, spliced images correspond to the representative blots shown in Figure 5e-h. **(B)** Membrane probed for β-tubulin (loading control). Lane assignments for all membranes are identical: **Lane 1-2, 6-7, 11-12**: Control (Ctrl) sample; **Lane 3-4, 8-9, 13-14**: CAPG-overexpression (*Capg*-OE) sample; **Lane 5, 10, 15**: Protein molecular weight marker. **(C)** Membrane probed for CAPG. **(D)** Membrane probed for PPARγ.

**Figure 5i**

**
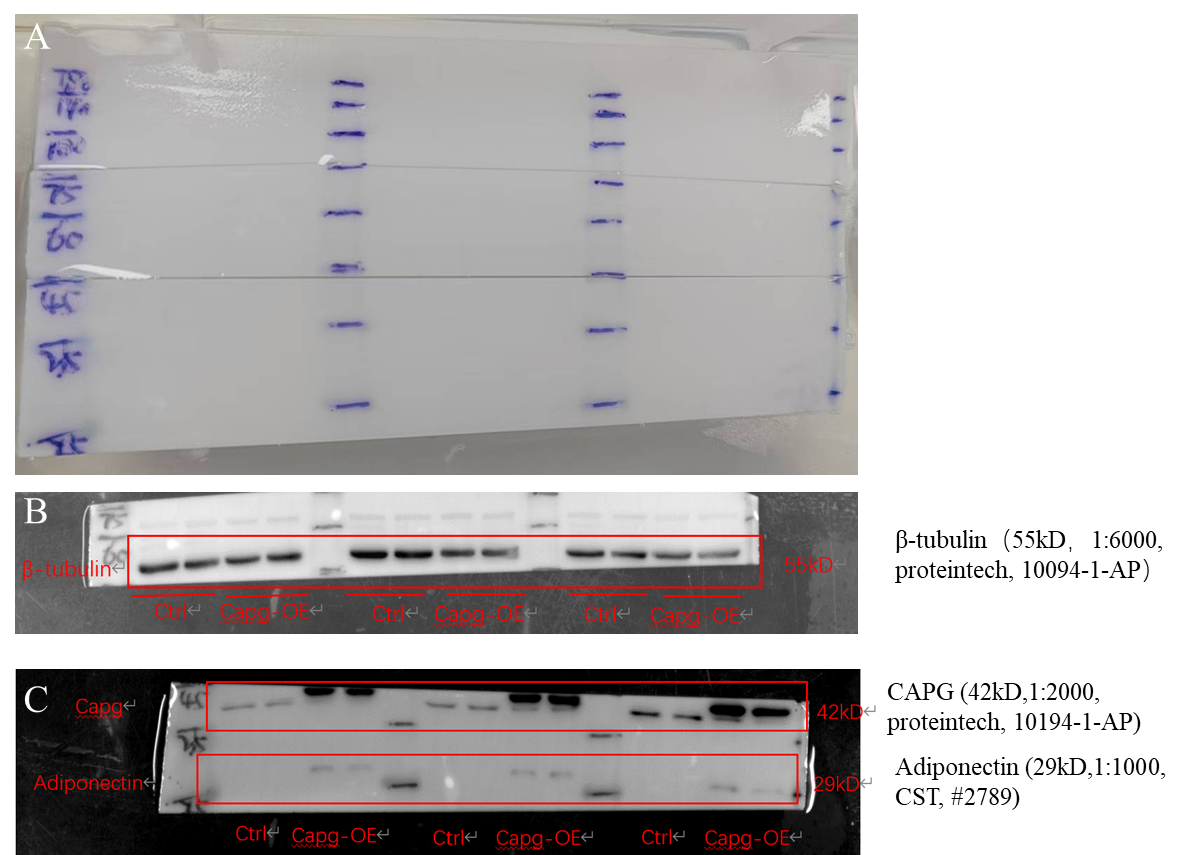
**

**Supplementary Figure S5. Raw Western Blot Images of Western blots shown in Figure 5i**

**(A)** These full, spliced images correspond to the representative blots shown in Figure **5i**. **(B)** Membrane probed for β-tubulin (loading control). Lane assignments for all membranes are identical: **Lane 1,6,11**: Protein molecular weight marker; **Lane 2-3, 7-8, 12-13**: Control (Ctrl) sample; **Lane 4-5, 9-10, 14-15**: CAPG-overexpression (*Capg*-OE) sample. **(C)** Membrane probed for CAPG and Adiponectin.
