# Supplementary material for: Capg enhances proliferation, adipogenesis, and inflammatory response in preadipocytes: insights from bioinformatics analysis and functional validation
Source: PeerJ. 2026 Feb 10;14:e20730. doi: 10.7717/peerj.20730 (PMC12903893; doi:10.7717/peerj.20730)
Supplement: Supplemental Information 4 — The raw quantitative data and statistical calculations for all key functional assays presented in the manuscript. The data is organized into separate sheets as detailed below. Sheet 1: CCK-8 Assay:Raw absorbance values (450 nm) and calculated cell viability/proliferation data for Control (Ctrl) and CAPG-overexpression (Capg-OE) groups over a time course. Sheet 2: Oil Red O Quantification:Raw data from the elution and spectrophotometric measurement of Oil Red O stain, quantifying total lipid content in differentiated adipocytes. Sheet 3: Oil Red O Positive Area Analysis (Microscopy):Quantitative image analysis data measuring the area of lipid droplets (Oil Red O positive regions) from multiple microscopic fields. Sheet 4-6: ELISA for Inflammatory Cytokines:Raw concentration values (pg/mL) for the pro-inflammatory cytokines MCP-1(Sheet 4), IL-6(Sheet 5), and TNF-α (Sheet 6) measured in cell culture supernatants. All statistical comparisons between the Ctrl and Capg-OE groups were performed using a two-tailed Student’s t-test. Data are presented as mean ± standard deviation (SD). [file peerj-14-20730-s004.docx]

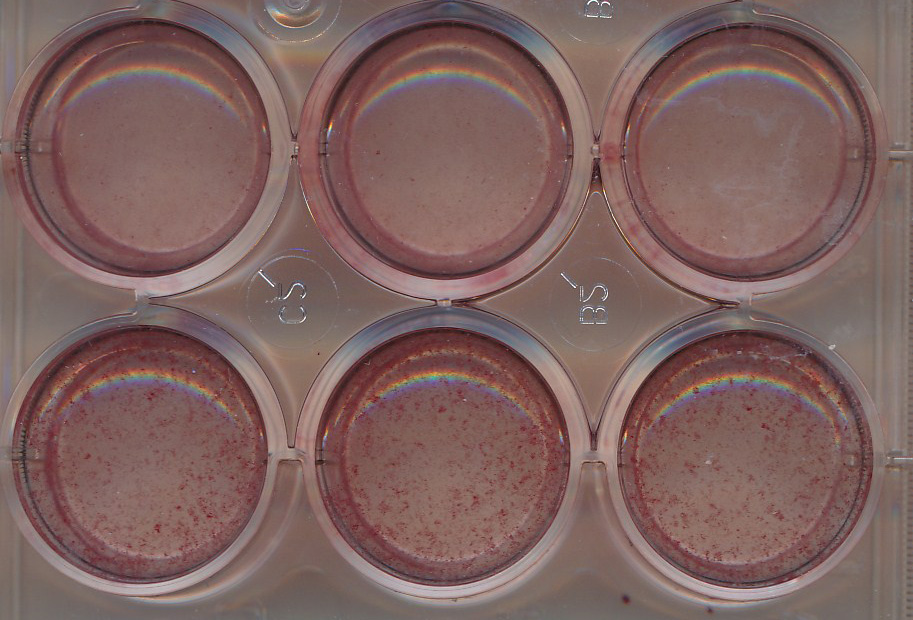


**Supplementary Figure S1. CAPG overexpression robustly enhances lipid accumulation in 3T3-L1 adipocytes.**
A representative multi-well plate showing Oil Red O staining on day 8 of differentiation. The top row shows control (Ctrl) groups, and the bottom row shows CAPG-overexpressing (*Capg*-OE) groups. The uniformly and intensely stained *Capg*-OE wells provide a clear visual demonstration of the pro-adipogenic effect of CAPG, consistent with the quantitative data presented in the main text.

**Supplementary Figure S2. Representative methodology for quantitative analysis of lipid accumulation.**
Shown are representative images of Oil Red O staining from a single well of the control (Ctrl) group, captured at 100× magnification. Five fields of view were randomly selected and imaged per well following this methodology. These images exemplify the baseline level of adipogenesis against which experimental groups were compared and served as the raw material for the subsequent quantification of lipid droplet-positive area.

**Supplementary Figure S3. Second control replicate confirming reproducible baseline.**
Representative images from a second control well (100× magnification), further confirming the consistent and low level of lipid accumulation in control cells throughout the study.

**Supplementary Figure S4. Third control replicate confirming reproducible baseline.**
Representative images from a third control well (100× magnification), further confirming the consistent and low level of lipid accumulation in control cells throughout the study.

**Supplementary Figure S5. CAPG overexpression enhances lipid accumulation in adipocytes.**
Representative Oil Red O staining images from the first replicate well of the CAPG-overexpressing (*Capg*-OE) group, captured at 100× magnification. These images provide direct visual evidence of increased lipid droplet formation upon CAPG overexpression, which served as the basis for quantitative analysis. When compared to the control replicates (Supplementary Figures S2-S4), the heightened staining intensity in this well is readily apparent, corroborating the pro-adipogenic role of CAPG.

**Supplementary Figure S6. Second replicate of enhanced lipid accumulation in *Capg*-OE cells.**
Representative images from a second *Capg*-OE well (100× magnification), confirming the pro-adipogenic phenotype.

**Supplementary Figure S7. Third replicate of enhanced lipid accumulation in *Capg*-OE cells.**
Representative images from a third *Capg*-OE well (100× magnification). The consistent results across all three replicates (Figs. S5-S7) provide robust support for the conclusion that CAPG promotes adipogenesis.
