# Supplementary material for: Capg enhances proliferation, adipogenesis, and inflammatory response in preadipocytes: insights from bioinformatics analysis and functional validation
Source: PeerJ. 2026 Feb 10;14:e20730. doi: 10.7717/peerj.20730 (PMC12903893; doi:10.7717/peerj.20730)
Supplement: Supplemental Information 6 [file peerj-14-20730-s006.docx]

**Line 55, 176**先去网页确定是否是表达芯片数据，不是的话不能用本流程。
First, verify on the website whether it is expression microarray data. If not, this workflow cannot be used.

**Line 61, 182**提取表达矩阵exp
Extract the expression matrix exp.

**Line 67, 188**提取临床信息
Extract clinical information.

**Line 70, 191**让exp列名与pd的行名顺序完全一致
Ensure the column names of exp completely match the row names of pd in order.

**Line 73, 194**提取芯片平台编号
Extract the microarray platform number.

**Line 95**差异分析，用limma包来做
Perform differential analysis using the limma package.

**Line 96**需要表达矩阵和Group，不需要改
Requires expression matrix and Group; no changes needed here.

**Line 103**为deg数据框添加几列
Add several columns to the deg data frame.

**Line 104, 219**加probe_id列，把行名变成一列
Add a probe_id column, converting row names into a column.

**Line 107, 222**加上探针注释
Add probe annotations.

**Line 109, 224**其他去重方式在zz.去重方式.R
Other deduplication methods are in zz.去重方式.R.

**Line 113, 228**加change列,标记上下调基因
Add a change column to label up-/down-regulated genes.

**Line 120, 235**加ENTREZID列，用于富集分析（symbol转entrezid，然后inner_join）
Add an ENTREZID column for enrichment analysis (convert symbol to entrezid, then perform inner_join).

**Line 131**其他物种[http://bioconductor.org/packages/release/BiocViews.html#___OrgDb](http://bioconductor.org/packages/release/BiocViews.html#___OrgDb)
For other species: <http://bioconductor.org/packages/release/BiocViews.html#___OrgDb>

**Line 172**实战代码有很多注意事项， 请不要不听课直接跑代码。
There are many important notes in the practical code. Please do not run the code without attending the lecture.

**Line 173**数据下载
Download data.

**Line 206**需要把Group转换成因子，并设置参考水平，指定levels，对照组在前，处理组在后
Convert Group into a factor, set the reference level, specify levels with the control group first and the treatment group second.

**Line 251**火山图----
Volcano plot.

**Line 291**工作路径，可以修改，可以设置为数据存放路径
Working directory; can be modified or set to the data storage path.

**Line 297**读取基因表达矩阵，矩阵是FPKM类型的数据
Read the gene expression matrix; the matrix contains FPKM-type data.

**Line 306**如果第一行是ID命名，就写成fpkm$ID，是不是基因名
If the first column is named ID, write as fpkm$ID. Check whether it contains gene names.

**Line 315**接下来要筛选出来表达缺失值，并移除
Next, identify and remove missing expression values.

**Line 328**过滤标准，可以修改
Filtering threshold; can be adjusted.

**Line 339**聚类
Clustering.

**Line 354**剪切高度不确定，故无红线h是调整点
The cut height is not fixed, hence no red line; h is the adjustment point.

**Line 357**所以这一步不一定能够做，剪切高度问题,这个根据实际设置后可用
This step may not always be feasible due to cut height issues; adjust based on actual situation.

**Line 358**这部分用于剪切后的样本剔除，调整高度的位置
This part is used for sample removal after cutting; adjust the height position.

**Line 374**载入性状数据
Load trait data.

**Line 507**剪切高度可修改
The cut height can be modified.

**Line 622**进行这部分计算必须每个模块内基因数量大于2，由于前面设置了最小数量是30，这里可以不做这个判断，但是grey有可能会出现1个gene,它会导致代码运行的时候中断，故设置这一步
This calculation requires more than 2 genes per module. Since a minimum of 30 was set earlier, this check may be omitted. However, the “grey” module might contain only 1 gene, which could interrupt the code execution, hence this step is included.

**Line 669**首先计算模块特征值(module eigengenes)
First, calculate module eigengenes.

**Line 672**计算module membership即MM
Calculate module membership (MM).

**Line 676** datTraits为目标性状的矩阵文件
datTraits is the matrix file for target traits.

**Line 678**提取每个模块的hubgene
Extract hub genes for each module.
